# Treatment of a Periodontic-Endodontic Lesion in a Patient with Aggressive Periodontitis

**DOI:** 10.1155/2016/7080781

**Published:** 2016-06-23

**Authors:** Mina D. Fahmy, Paul G. Luepke, Mohamed S. Ibrahim, Arndt Guentsch

**Affiliations:** ^1^Department of Surgical Sciences, Marquette University School of Dentistry, Milwaukee, WI 53233, USA; ^2^Department of Endodontics, Faculty of Dentistry, Mansoura University, Mansoura 35516, Egypt; ^3^Center of Dental Medicine, Jena University Hospital, Friedrich-Schiller-University, An der Alten Post 4, 07743 Jena, Germany

## Abstract

*Case Description*. This case report describes the successful management of a left mandibular first molar with a combined periodontic-endodontic lesion in a 35-year-old Caucasian woman with aggressive periodontitis using a concerted approach including endodontic treatment, periodontal therapy, and a periodontal regenerative procedure using an enamel matrix derivate. In spite of anticipated poor prognosis, the tooth lesion healed. This case report also discusses the rationale behind different treatment interventions.* Practical Implication*. Periodontic-endodontic lesions can be successfully treated if dental professionals follow a concerted treatment protocol that integrates endodontic and periodontic specialties. General dentists can be the gatekeepers in managing these cases.

## 1. Introduction

Decision-making processes of a tooth as having a good, questionable (but treatable), or hopeless prognosis (with extraction required) are based on periodontal, endodontic, and restorative parameters [1]. The periodontal classification of teeth is based on the amount of attachment loss and probing pocket depth or furcation involvement [2]. Besides the periodontal parameters, predisposing factors for tooth loss in patients with periodontitis are the presence of pulpal infection/necrosis and caries [3]. However, recent research has demonstrated that, even with periodontic-endodontic involvement, teeth regarded as hopeless can be successfully treated [4].

The interrelationship between the periodontium and pulp was first described by Simring and Goldberg in 1964 [5]. Simon and colleagues noted that combined periodontic-endodontic lesions are composed of an endodontically induced periapical lesion on a tooth that is also periodontally compromised [6]. Communication exists between the periodontium and the pulpal tissues by means of canals. Langeland and colleagues discussed how pulpal inflammation from involved lateral canals or root caries causes damage to the pulp [7]. Thus, the extension of pulpal infections to the periodontium and vice versa may be attributed to these canals [8]. In animal studies, there is a high predominance of lateral canals in posterior teeth that communicate with the floor of the pulp and the periodontal ligament [9, 10]. Several other pathways which may act as potential facilitators of periodontally derived endodontic lesions have been noted in literature and include lingual grooves, root and tooth fractures, root anomalies, fibrinous communications, and trauma induced root resorption [11]. Where a periapical infection and/or inflammation exist, the periodontium can be significantly damaged. However, following proper root canal therapy (RCT), healing occurs without a residual effect [12]. Clinical presentation of periodontic and endodontic abscesses may bear close similarities, although differing in their point of origin. Combined periodontic-endodontic lesions occur as a result of the interaction between their respective disease origins on the same tooth, irrespective of the sequence in which the diseases occur [8]. Differential diagnoses and treatment methods are partially dependent on the evaluation of pulp vitality [12]. If periodontal pockets exist, but the pulpal tissue reaction is normal, then either the acute or the chronic inflammation is of periodontal origin. However, when the pulp is found to be nonvital, the inflammatory process passing through the lateral canals or apical foramen causing a lesion in the periodontium is of endodontic origin [8]. When an infection and/or inflammation are evident within the pulp, with periodontal disease that was preexisting, the pulpitis may be considered secondary to the periodontal disease. Importantly, the existence of subgingival calculus and the intensity and location of inflammation both aid in determining the primary source of the disease [8, 13]. Evidently, combined pulpal and periodontal issues account for more than 50% of tooth mortality [14]. In addition, several studies have indicated that combined periodontic-endodontic therapy is imperative for successful healing of such a combined lesion [13, 15] although the primary source of combined lesions is rarely precisely identified. This case report aims to illustrate a significant clinical case and a suggested evidence-based treatment protocol for periodontic-endodontic lesions, which allows for maintaining teeth that may be considered hopeless.

## 2. Case Presentation

A 35-year-old Caucasian female was referred to a periodontist, after a diagnostic periapical radiograph of tooth 36 (lower left first molar) at the general dentist's office showed vertical bone loss extending to the apex of the distal root.

**Figure 1:**
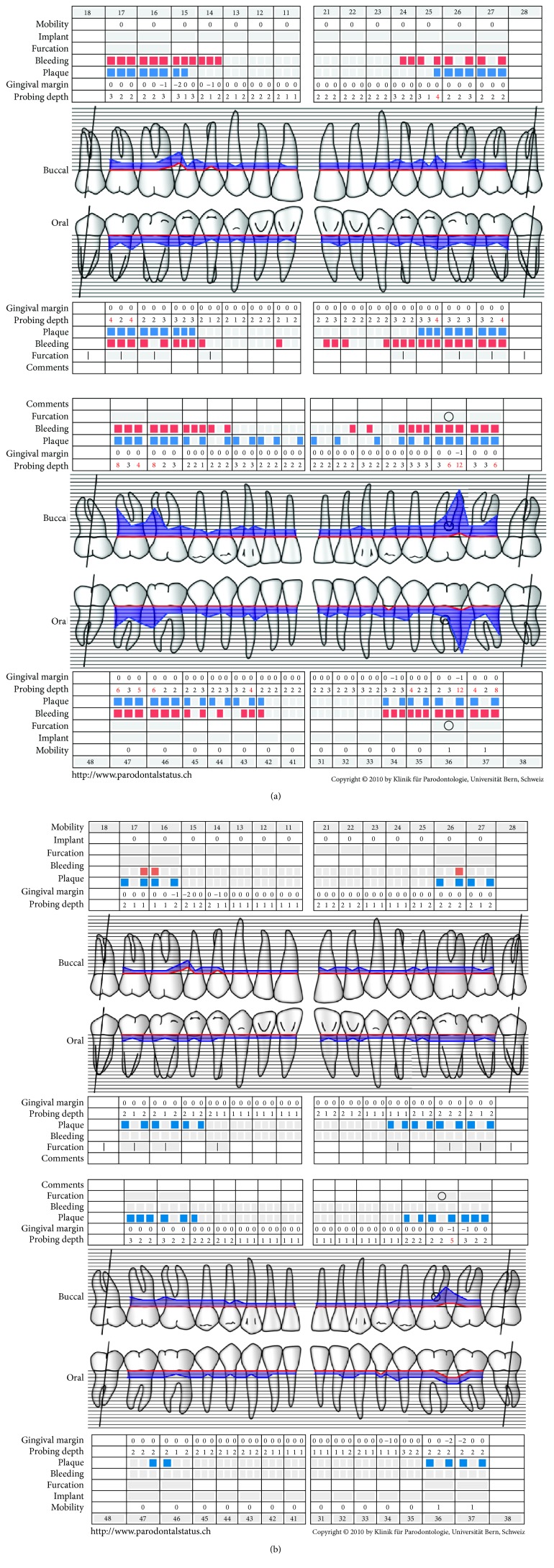
Periodontal charting of the initial visit (a) and 24 months (b) after active periodontal treatment. The probing depths distal (buccal and oral) of tooth 36 were reduced from 12 mm to 4 mm. The mean CAL was reduced from 2.7 mm to 1.5 mm, while bleeding on probing and plaque level were reduced from 55% to 1% and 48% to 20%, respectively.

The patient was generally in good health with good oral hygiene (Figure 1). She had never smoked and she routinely visited her general dentist for annual oral exams. The clinical examination demonstrated increased periodontal probing depths up to 12 mm on the distal root surface of tooth 36 and up to 8 mm on the mesial root surface of tooth 37, as well as 8 mm between teeth 46 and 47. Tooth 36 presented class 1 furcation involvement lingually. All teeth responded normally to cold and electric pulp testing (EPT), except tooth 36 which showed a delayed response and was diagnosed with asymptomatic irreversible pulpitis with asymptomatic lesion of endodontic origin. Radiographic examination revealed vertical bone loss on the distal root surface of tooth 36 extending to the root apex and alongside the mesial wall of the distal root and alveolar bone loss between teeth 25 and 26. The microbiological testing of the subgingival biofilm [16] resulted in the presence of* Aggregatibacter actinomycetemcomitans*,* Porphyromonas gingivalis*,* Prevotella intermedia*,* Tannerella forsythia*, and* Treponema denticola* (bacterial load ≧ 10^5^). The periodontal diagnosis was aggressive periodontitis with a combined periodontic-endodontic lesion (primary periodontal origin) at tooth 36. Possible treatment interventions for tooth 36 were explained to the patient, including (1) extraction, ridge augmentation, endosseous implantation, and implant-supported crown, (2) extraction and fixed partial denture, and (3) endodontic and periodontal treatment to retain the tooth. The patient requested to “save the tooth” and opted to have endodontic and periodontal treatment.

The treatment of tooth 36 followed a concerted protocol, which included endodontic and periodontal treatment steps (Figure 5). The goal of the anti-infective therapy (phase 1 therapy) was to reduce the bacterial load and inflammation. The patient underwent an oral prophylaxis session including individualized oral hygiene instructions. RCT was initiated immediately [17] and performed using an operating microscope (OPMI pico, Carl Zeiss AG, Jena, Germany) by an endodontist. Root canal treatment was performed in 2 visits; on the first visit, canals patency was achieved using #10 K hand files. The pulp tissue in the distal canal appeared necrotic, while in the mesial canals the tissue was bleeding, which can be interpreted as signs of vitality and/or pulpitis. The working length was established with a Raypex apex locator (VDW, Munich, Germany). The mesial canals were instrumented with the Mtwo rotary system up to size 30, 0.05 taper (VDW, Munich, Germany), while the distal canal was instrumented until size 40, 0.04 taper. All canals were irrigated with 5.25% sodium hypochlorite. Canals were dried with sterile paper points and dressed with calcium hydroxide (UltraCal XS, Ultradent, South Jordan, USA) for seven days and the tooth was restored with composite resin. At the second visit, the tooth was reaccessed and calcium hydroxide was removed using hand files and irrigation with sodium hypochlorite. The canals were irrigated with 17% EDTA liquid and 5.25% sodium hypochlorite; both were activated with EndoActivator (Dentsply Tulsa Dental Specialties, Tulsa, OK). Canals were dried and obturated using the Element Obturation Unit (SybronEndo, Orange, CA, USA). Finally, a composite reconstruction was performed.

Shortly after cleaning and shaping of the root canals during root canal therapy, the nonsurgical periodontal treatment was performed as full-mouth scaling and root planing (SRP) within 24 hours, using ultrasonic and manual instruments [18]. Systemic antibiotics (amoxicillin 500 mg three times a day and metronidazole 400 mg three times a day for 8 days) were prescribed as a consequence of the diagnosis of aggressive periodontitis [19] and the subgingival microbial profile [19, 20].

**Figure 2:**
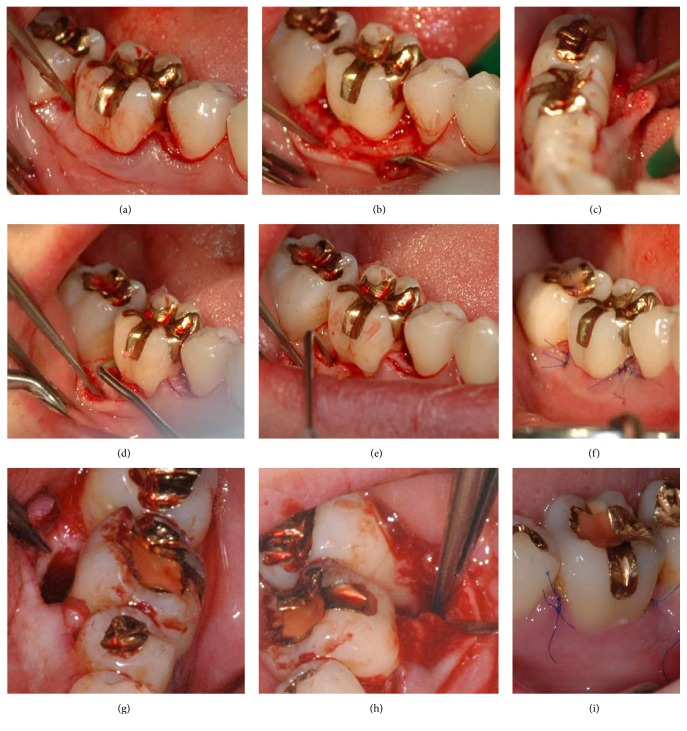
Microsurgical access flap and use of enamel matrix derivate to treat the defects at tooth 46 (a–f) and tooth 36 (g–i). After crevicular incision (a), the papillae were preserved and the flaps reflected buccally (b and h) and lingually (c and g) to gain access to the defect. The granulation tissue was removed and the root surfaced planed (d) and prepared (PrefGel, Straumann, Freiburg, Germany) before the enamel matrix derivative (Emdogain, Straumann, Freiburg, Germany) was applied (e). Photographs (f) and (i) show the primary wound closure, immediately after the surgery (f) and one week postoperatively (i).

Regenerative periodontal therapy using a biological factor (Emdogain, Straumann, Freiburg, Germany) was performed 4 weeks after anti-infective therapy—first at the lower right site, followed by the lower left site (Figure 2). This short time span between nonsurgical and surgical corrective periodontal treatment was chosen with respect to the severe attachment loss and the combined periodontic-endodontic lesion and to reduce the risk of reinfection of a potentially residual pocket [21]. The root canals were filled with gutta-percha and AH-plus (Dentsply DeTrey, Konstanz, Germany) on the same day before surgical access to the periodontic-endodontic lesion. The regenerative therapy was performed as a microsurgical access flap with preservation of the papilla soft tissue using a technique described by Wachtel et al. [22] in conjunction with use of an enamel matrix derivative [23].

**Figure 3:**
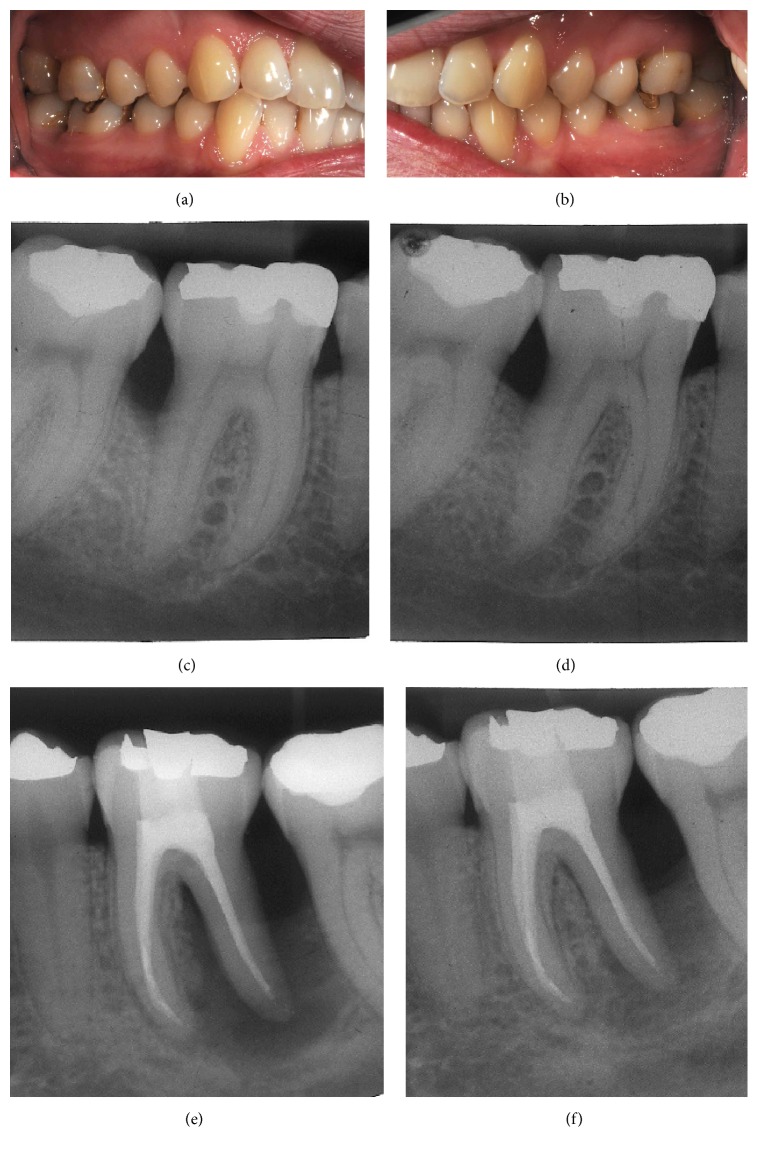
Clinical photographs (a, b) and radiographic images of teeth 46 (c, d) and 36 (e, f) at the 6- month reevaluation. Periodontal defects on both teeth demonstrated radiographic gain of bone structure in comparison to the baseline visit (c, e) 6 months after regenerative periodontal treatment (d, f).

**Figure 4:**
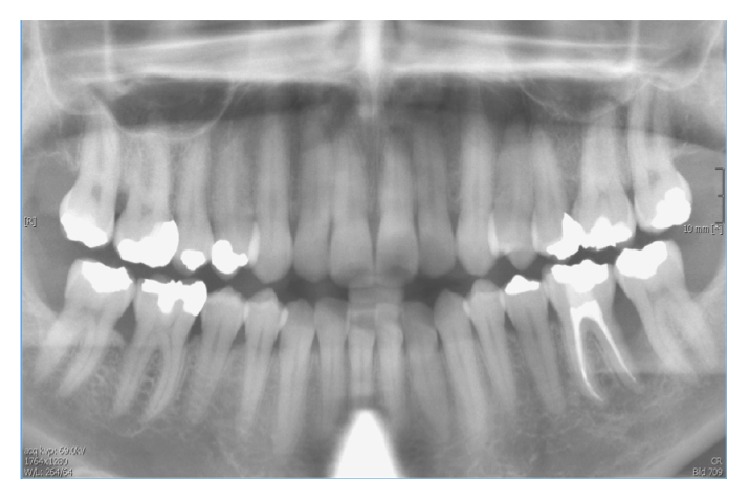
Panoramic radiograph. The periodontal defects, especially at tooth 36, showed progressive periodontal healing 24 months after regenerative periodontal treatment.

**Figure 5:**
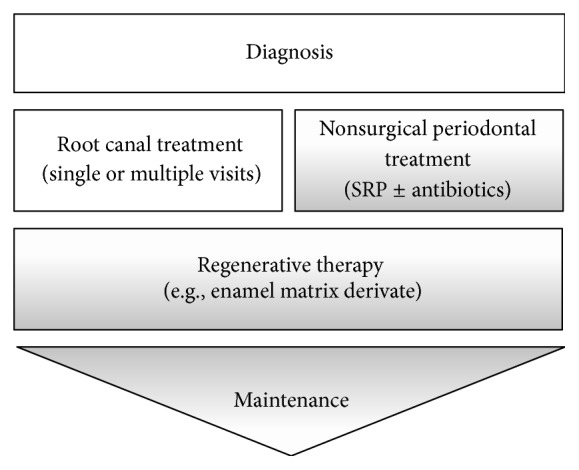
Concerted treatment steps for the treatment of teeth with periodontic-endodontic lesions. Following diagnosis, root canal treatment and nonsurgical periodontal treatment should be completed within 4 weeks. Following nonsurgical/RCT, regenerative therapy should be completed within 4–12 weeks. Maintenance is lifelong.

The microbial examination six months after active periodontal treatment was only positive for* Treponema denticola* with a bacterial count less than 10^3^; all other investigated periodontopathogens were not detectable. Clinical and radiographic measurements at tooth 36 suggested regeneration of the periodontal structures with a gain clinical attachment of 9 mm (distobuccal) and 8 mm (distolingual), respectively (Figure 3). The healing of the periodontic-endodontic lesion showed long-term stability (Figure 4).

## 3. Discussion

This case report of a patient with a periodontic-endodontic lesion demonstrates that retaining a tooth with a poor prognosis is possible when the treatment follows a structured and interdisciplinary approach. Basic requirements leading to the decision to save rather than extract the tooth were the good oral hygiene and compliance of the patient [24] as well as the restorability of the tooth [1]. Treatment alternatives or options such as extraction followed by (i) augmentation, implantation, and implant-supported crown or (ii) fixed partial dentures were discussed with the patient. A closer look at the clinical and radiographic findings and at the available evidence led to the conclusion that these treatment options may not be the most suitable and most effective at treating the periodontic-endodontic lesion.

A systematic review compared the long-term outcome of RCT and restoration with implant-supported single crowns (ISC) and fixed partial dentures (FDP) and identified that RCT and ISC resulted in superior-long-term survival, compared to the FDP [25]. A recent analysis by Setzer and Kim demonstrated that while the survival rates of endodontically treated teeth and implants are comparable, the success rates may not be. After 7 to 9 years, the success rate for implants was 74% while for endodontically treated teeth it was 84%. In addition, the implant group had a significantly higher rate of complications. Further, In 17.9% of the implant cases versus only 3.6% of the endodontic cases, survival occurred because complications were treated [26]. Peri-implant diseases are the major biological complication in implants [27]. The history of periodontal disease should be considered a risk-factor for future peri-implant disease [28].

The diagnosis and classification of periodontal diseases are almost entirely based on traditional clinical assessments, for example, (i) presence or absence of clinical signs of inflammation, (ii) probing depths, (iii) extent and pattern of clinical attachment loss and bone, (iv) patient's medical and dental history, and (v) presence or absence of miscellaneous signs [29]. Additional essential components for diagnosis are intraoral radiographs, such as periapical radiographs and horizontal and vertical bitewings [29]. Three-dimensional-imaging of the defect anatomy in the described case would be for scientific interest to attain additional information about the associations between the clinical success and the size and configuration of the defect. However, CBCTs should only be used when the need for imaging cannot be satisfied adequately by lower dose conventional dental radiography or alternate imaging modalities [30].

The goal of the anti-infective therapy was to suppress the bacterial load and to establish a balance between bacterial burden and host response to allow for healing to occur.

Therefore, due to the periodontic-endodontic lesion at the lower left first molar, it was imperative that the therapy regimen include endodontic treatment. Vakalis and collaborators demonstrated that RCT followed by nonsurgical periodontal treatment can be very effective and result in the improvement of clinical parameters together with alveolar bone gain [31].

Cortellini et al. [4] demonstrated that regenerative periodontal treatment is effective even in hopeless teeth and may therefore be an alternative to extraction. Imbedded in a consequent and structured maintenance system in addition to good compliance on the part of the patient, questionable and hopeless teeth can be retained over an extended period of time [32]. To maintain the treatment outcome, several studies have shown that patients who comply with regular periodontal maintenance visits experience less attachment loss and lose fewer teeth than patients who receive less periodontal maintenance [33–36]. Axelsson et al. demonstrated that the periodontal status can be maintained over long periods [24]. In addition to these findings, the periodontal regenerative therapy of furcation-involved teeth seems to be more cost-effective than extraction and replacing the tooth with an implant [37].

Regenerative periodontal treatment can be performed as guided tissue regeneration (with and without bone graft materials) as well as using biological factors such as an enamel matrix derivate. Both methods result in a comparable clinical outcome [23], but enamel matrix derivatives offer less discomfort for the patient and show less postoperative complications [38]. The use of an enamel matrix derivative has been shown to significantly improve probing attachment levels (1.1 mm) and reduce pocket depths (0.9 mm) when compared to a placebo or control as discussed by Esposito and colleagues [39] and is also effective in treatment of class 1 and 2 furcation defects [40].

## 4. Conclusions and Practical Implication

This case report demonstrates that a concerted interdisciplinary approach can result in improving and maintaining the natural dentition in order to achieve health, comfort, esthetics, and function [41] even in teeth with periodontic-endodontic lesions with primary periodontal origin. The treatment should follow a suggested protocol, which starts with an oral prophylaxis session (oral hygiene instruction and supragingival scaling), immediately followed by RCT of the affected tooth. Subsequently, the nonsurgical periodontal treatment (SRP), which may include the application of adjunctive antibiotics, is completed. Anti-infective treatment and periodontal regenerative therapy can then be performed to guide the wound healing towards regeneration of lost periodontal structures. Further research, especially clinical trials, is needed to evaluate the suggested treatment approach or alternative options.
